# Prediction of gap balancing based on 2-D radiography in total knee arthroplasty for knee osteoarthritis patients

**DOI:** 10.1186/s42836-023-00218-y

**Published:** 2023-11-16

**Authors:** Zhuo Zhang, Yang Luo, Chong Zhang, Xin Wang, Tianwei Zhang, Guoqiang Zhang

**Affiliations:** 1 Department of Adult Reconstruction and Joint Replacement, Senior Orthopedic Department, Fourth Medical Center, Chinese PLA General Hospital, No. 51, Fucheng Road, Beijing, 100048 China; 2 Department of Orthopedics, First Medical Center, Chinese PLA General Hospital, No.28, Fuxing Road, Beijing, 100853 China; 3 Yunnan Baiyao Group Medicine Electronic Commerce Co., Ltd, No. 3686 Yunnan Baiyao Street, Chenggong District, Kunming, 650500 Yunnan China

## Abstract

**Background:**

To investigate the influence of osteophytes on postoperative gap balancing, and to work out a predictive model of the relationship between osteophyte size and gap gaining in primary total knee replacement.

**Methods:**

One hundred and ten patients were enrolled in the study. Pre- and postoperative radiographs were collected and analyzed. They were assigned to the training dataset and test dataset randomly at a ratio of 9:1 by using the statistical package R (version 4.0.5). Size and marginal distances of osteophytes, planned bone cut planes, predicted bone cuts and joint gaps were labeled on the preoperative standing anteroposterior and lateral views, while actual bone cuts and joint gaps were recorded on the postoperative plain films, respectively. Statistical analysis was performed.

**Results:**

Actual joint gaps were significantly related to the distances of medial and lateral predictive bone cutting lines, bone cut thickness on tibial side and posterior condylar, as well as size and marginal distances of osteophytes (*P* < 0.05). A predictive equation was generated, with a root mean square error (RMSE) of 3.4761 in validation. A 2-D planning system with adjustable input parameters and dim predictive outputs on joint gap was developed. The equation is $S \left(Joint Gap\right)=1.82+0.15*y+0.552*Tibial cut+0.953*Femoral cut+0.197*Post Condyle$

**Conclusion:**

Postoperative joint gap can be predicted on the basis of preoperative measurements on 2-D plain films. Larger sample size may help improve the effectiveness and accuracy of the predictive equation.

## Background

Total knee arthroplasty (TKA) is commonly used for surgical treatment of knee osteoarthritis (OA) with good to excellent results reported. Preoperative planning is essential for a successful TKA procedure. The precision of planning has improved with the utility of digital planning systems and applying planning on three-dimensional images, which are considered more accurate [1].

As one of the basic steps of TKA, gap balancing, an important technique, can create stable and balanced medial–lateral and flexion–extension gaps. Gap balancing can be variously achieved by removal of osteophytes, proper release of soft tissues, and reasonable compromise to alignments, depending on different alignment concepts adopted. Intraoperatively, gap balance is a hand-check procedure, that relies on the experience of surgeons. Although the effect of osteophyte removal can be predicted from preoperative radiographic films, it is difficult to precisely quantify a balanced gap after release.

Computer science has been increasingly used in various medical settings. More recently, artificial intelligence (AI)-based medical systems have become commercially available. Modern systems armed with machine learning algorithms have been applied in image processing, health state monitoring and prediction, detection of diseases, medication administration and management of patients, among others [2–8].

In this study, we attempted to develop a 2-D planning system with an algorithm of the balanced gap from the preoperative plain film of the knee. Computer-assisted methods were used to investigate the influence of osteophytes on postoperative gap balancing and to obtain an equation that predicts the relationship between osteophyte size and gap gaining in primary TKA for OA.

## Methods

### Sample enrollment

**Fig. 1:**
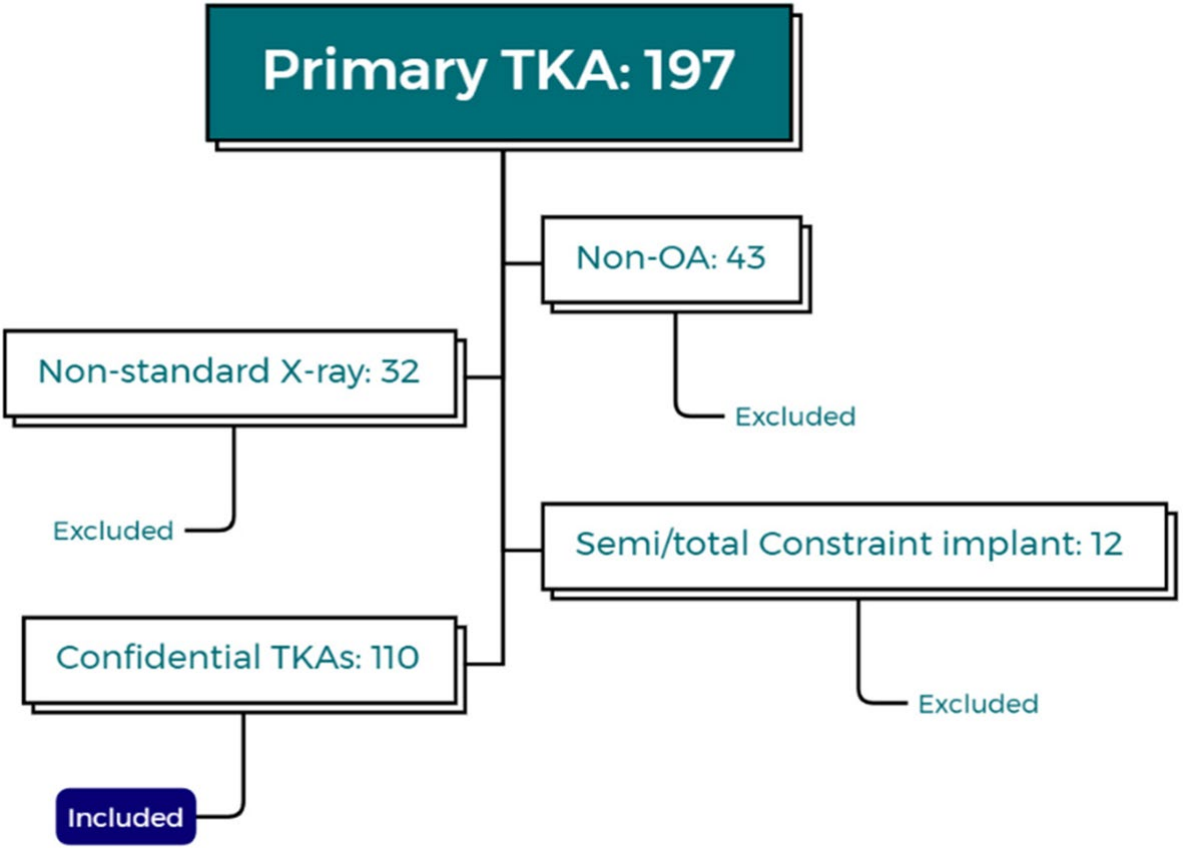
Criteria for inclusion and exclusion. Non-OA: rheumatic, post-traumatic or neoplasm diseases; Non-standard X-ray: significant rotation seen on anteroposterior view; Semi/total constraint implant: condylar constraint (CCK) or hinged implant

Radiographic data of patients who received primary TKA from January to December 2021 were reviewed. One hundred and ten TKA cases that met the inclusion criteria were enrolled. Criteria for inclusion and exclusion are shown in Fig. 1. Standard pre- and postoperative anteroposterior and lateral radiographs of the knee were collected for analysis. The study was approved by Ethics Committee of our institute (Approved Number: S2020-005–01).

### Radiographic labelling

**Fig. 2:**
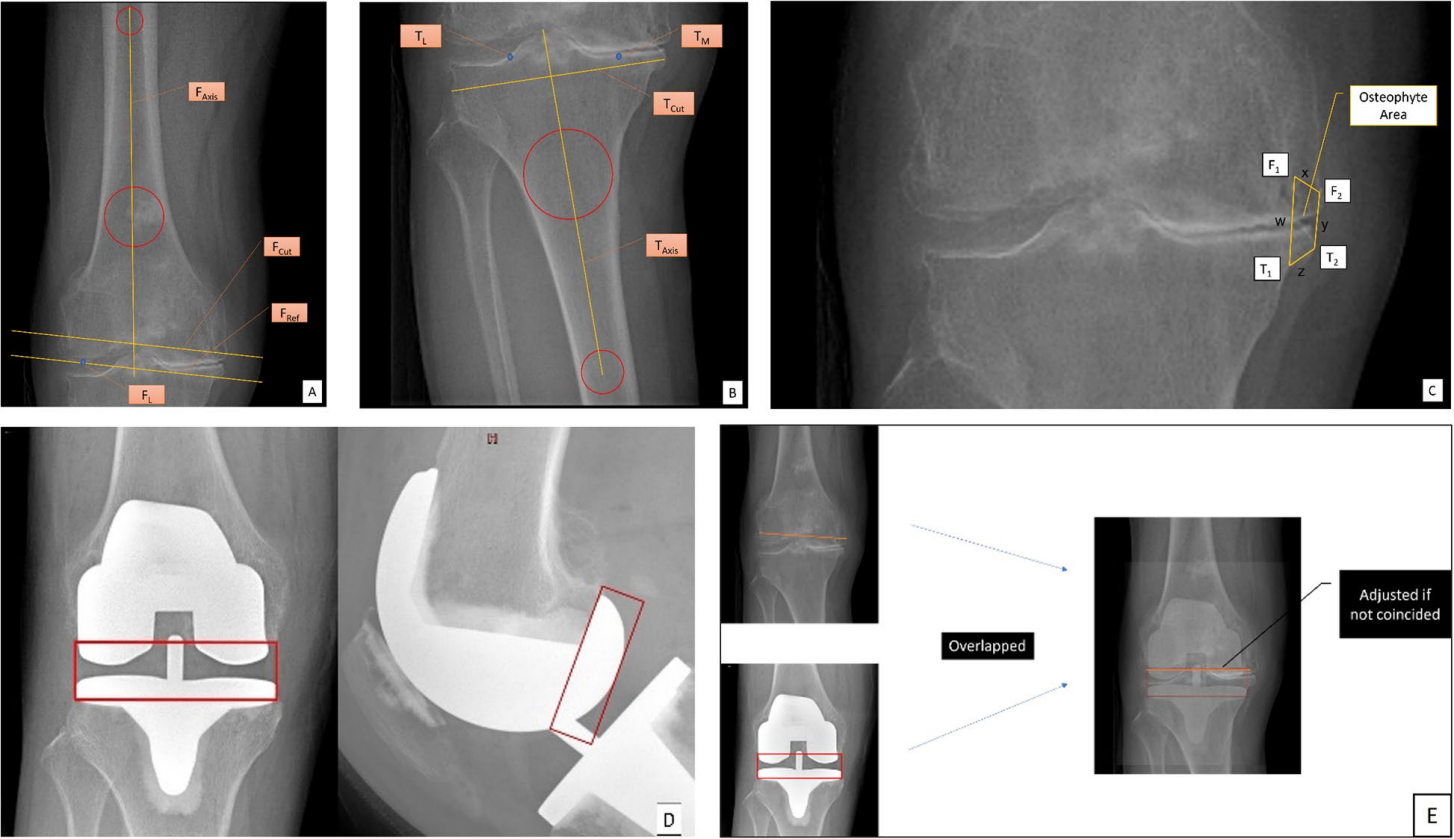
Radiographic labeling and calibration of preoperative and postoperative X-rays. Labels on AP view demonstrated elements defined in Table 1. **A** & **B**, preoperative X-ray. **C**, osteophyte area. **D**, postoperative X-ray. **E**, calibration of magnification

Several elements were defined on preoperative AP and lateral plain films (see Table 1, Fig. 2A and B). The thickness of the bone cut was calculated. Due to the irregularity in osteophyte shapes, the area of osteophytes on the femoral and tibial sides was taken as trapeziform, of which the apexes were defined on plain film. Borders of the trapeziform were designated w, x, y, and z (Fig. 2C).

**Table 1:** Definition of elements for measurement on preoperative plain films (Fig. 2A and B)

Elements in System	Description of computer algorithm	Function
Femoral canal/axis (F_Axis_)	A line connecting the centers of two incircles located at diaphysis and metaphysis of femur, respectively	Simulating intra-medullary rod of femoral cutting jig
Femoral cutting reference line (F_Ref_)	A line angulating 85° laterally to femoral canal/axis (aLDFA), in contact with distal margin of lateral/medial femoral condyle (height determined by the first contact with lateral or medial margin, which means the lower one in medial and lateral contact points were counted)	Simulating distal contact of cutting jig to distal femoral articular surface
Checkpoint for medial distal femur (F_M_)	Contact point of Line F_Ref_ and distal margin of medial femoral condyle	The ***lower*** point was be chosen as contact point
Checkpoint for lateral distal femur (F_L_)	Contact point of Line F_Ref_ and distal margin of lateral femoral condyle	
Initial distal femur cutting line (Line F_Cut_)	A line parallel to Line F_Ref_, being 9 mm apart proximally	Simulating 9-mm thickness of distal cut
Femoral cut	True distance from F_L_/F_M_ to F_Cut_	Lower point of F_L_/F_M_ was selected for calculation
Tibial canal (T_Axis_)	A line along with anatomic axis of tibial shaft	Anatomic axis of tibial shaft
Checkpoint for medial proximal tibia (T_M_)	Lowest point of medial tibial condylar	
Checkpoint for lateral proximal tibia (T_L_)	The point located laterally at 3/8 of the total width of tibial plateau	
Initial proximal tibial cutting line (Line T_Cut_)	A line perpendicular to tibial anatomic axis Distance from Point T_L_ to Line T_Cut_ was defined as 10 mm	Simulating 10-mm thickness of tibial cut
Tibial cut	True distance from T_L_ to Line T_Cut_	
Checkpoints for distal femoral osteophyte (F_1_ & F_2_)	F_1_: most proximal point of femoral osteophyte basement, defined as the turning point of cortex to the upper margin of femoral osteophyte F_2_: most protruding point of femoral osteophyte, defined as the turning point of femoral osteophyte margins	
Checkpoints for proximal tibial osteophyte (T_1_ & T_2_)	T_1_: most distal point of tibial osteophyte basement, defined as the turning point of tibial cortex to the lower margin of tibial osteophyte T_2_: most protruding point of tibial osteophyte, defined as the turning point of tibial osteophyte margins	
Osteophyte area (Trapeziform F_1_F_2_T_2_T_1_)	A trapeziform area formed by four check points of osteophytes

On the postoperative films, only bone-cut lines were located (Table 2). We assumed that all included cases achieved a balanced rectangular joint gap after bone cut and soft tissue balancing. A rectangle was defined, with its longer borders standing for bone cut lines, while the shorter borders used for the measurement of joint gap (Fig. 2D).

**Table 2:** Definition of elements for measurement on postoperative plain films (Fig. 2C)

Elements	Description	Function
Femoral cutting line	Backside of distal portion of femoral component	Femoral cutting line
Tibial cutting line	Backside of tibial component	Tibial cutting line
Joint space rectangle	Rectangle with femoral and tibial cutting lines as longer borders	Simulating parallel joint space achieved after bone cutting and soft tissue balancing
Joint gap	Shorter borders of joint space rectangle	Joint gap achieved
Posterior condylar rectangle	Rectangle with backside and tangent line to the posterior arc of posterior condyle of femoral implant as longer borders	
Thickness of posterior condyle (on lateral view)	Shorter borders of the posterior condylar rectangle	Thickness of posterior condyle

### Calibration of magnification and modification of labeled elements

Given errors that might occur in magnification, a marker was used during radiographic examination. Calibrated data were collected using unified magnification.

Since it was difficult to obtain the exact thickness of bone intraoperatively, and cutting lines on the femoral and tibial sides might not be precisely shown on X-ray films, modification of cutting line(s) might be required to obtain more precise parameters. Pre- and postoperative AP films were overlapped in a unified magnification. If cutting lines on pre- and postoperative films were not consistent, it was adjusted on preoperative AP film, and the amount of cutting was recalculated (Fig. 2E).

### Surgical technique

Gap balancing techniques were utilized during the TKA procedure [9]. Routine mid-incision and medial parapatellar approach were applied for arthrotomy. Osteophytes protruding from the medial cortex of the distal femur and proximal tibia were removed. Since osteophytes of the posterior distal femur were inaccessible before bone cutting, they were left intact until 4-in-1 cutting was performed. A distal femoral cut was made first, with valgus of cutting jig set to 5°. Afterwards, a perpendicular tibial cut was made. Two lamina spreaders were inserted into medial and lateral joint spaces, respectively. The lateral joint gap should be big enough to allow for spacer trial, while a 1-to-2 mm compromise of medial gap might be acceptable, because posterior osteophytes were left intact. External rotation of femoral component was determined with the knee in 90° flexion. Spreaders were inserted into the space between posterior condyles and tibial plateau, and maximum tension of collateral ligaments was achieved. Upon acheivement of balanced ligament tensions were achieved, a line parallel to tibial plateau was drawn on the surface of distal femur, which served as the external rotation reference. Then the four-in-one cutting procedure would be performed by following the principles of posterior reference procedure, as the distance between cutting lines of posterior condyles and tibia equaled to the total thickness of prosthesis in flexion. A spacer trial might be helpful for determining the position of cutting lines. As it lay on the tibial plateau, the upper side of the spacer stood for the position of cutting line of posterior femur. Osteophytes of posterior femur were removed. Flexion and extension gaps should be confirmed with a spacer or spreaders. We recommended using the trial spacers that go with the prosthetic system, or performing a trial reduction instead. No more soft tissue release should be performed as soon as bone cut is accomplished.

### Estimation of joint gap upon osteophyte removal and soft tissue release

**Fig. 3:**
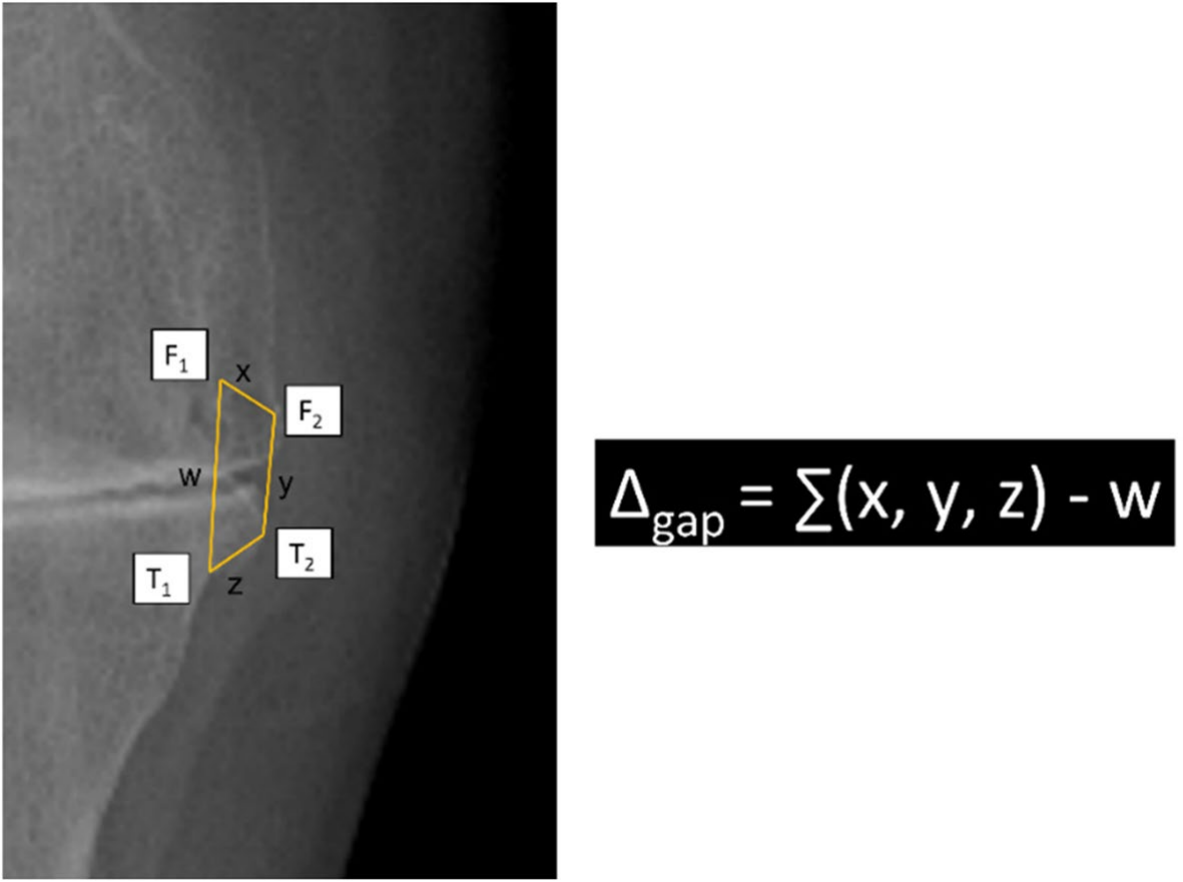
Calculation of gap after osteophyte release

According to the rationale of gap balancing technique in TKA, soft tissue release should be minimal. The additional gap resulted mainly from removal of osteophytes, and was calculated from the difference between the outer and inner rims of osteophyte trapeziform area (Fig. 3).

### Statistical analysis and predictive equation simulation

One hundred and ten cases were included in our study and were divided into a training dataset and a test dataset randomly at a ratio of 9:1. Statistical package R (Version 4.0.5, The R Foundation for Statistical Computing, Vienna, Austria) was used for statistical analysis. Relations among continuous variables were assessed in terms of Pearson’s correlation coefficient, while differences were tested by an independent *t*-test. A *P* < 0.05 was considered statistically significant.

Univariable linear regression analysis was performed to evaluate the association between each predictive variable and the joint gap. Additionally, stepwise regression was conducted. Considering the correlation between independent variables, independent variables that had significant influence on dependent variables and were independent of each other were chosen in the multivariable analysis. Meanwhile, distributions of residual and fitting values were checked to make sure that they were in line with the linear regression hypothesis.

A tenfold cross-validation was conducted to avoid a certain sampling bias. The fold with the best predictive effect was selected as the final model.

To compare the performance of the model and predict the contribution of the predictors in the multivariate linear regression model, we used R-squared to evaluate the performance of the models, with higher values indicating a more incredible prediction. Furthermore, the models' root mean square error (RMSE) and mean absolute error (MAE) of the models were also calculated [10]. Low values of RMSE and MAE indicated good predictive power of the mode.

## Results

### Data description

**Fig. 4:**
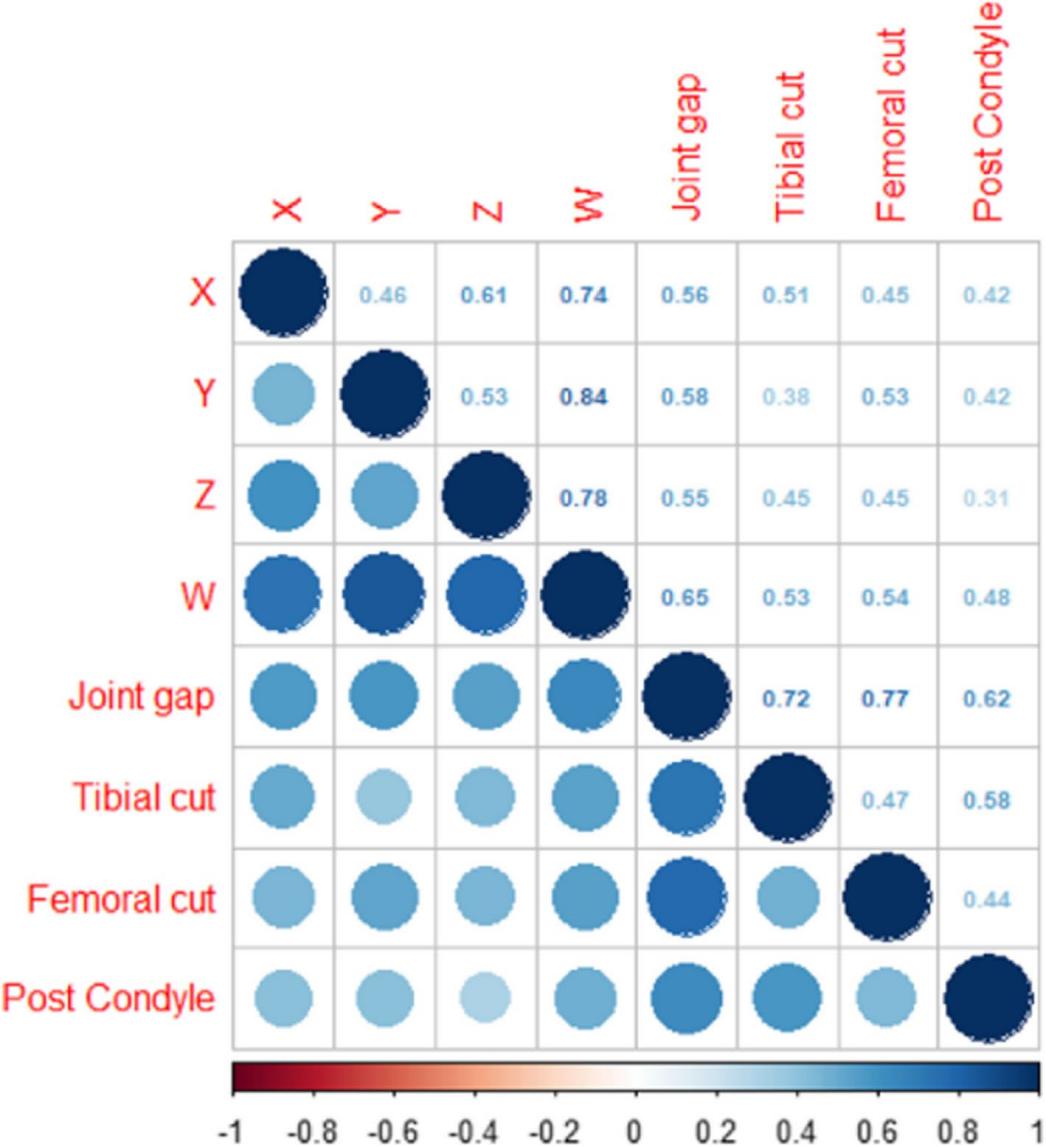
Correlation of continuous variables

All variables showed no significant differences in mean values between the training dataset and test datasets (Table 3). As shown in Figure, thickness of tibial cut, femoral cut and posterior condyle, length of borders of osteophyte trapeziform (w, x, y, and z) and joint gap were correlated significantly (*P* < 0.05). Moreover, border lengths of osteophyte trapeziform were correlated with each other, as well (Fig. 4).

**Table 3:** Description and differences between training dataset and test dataset

Variables	All (*n* = 110)	Training (*n* = 99)	Test (*n* = 11)	*P*-value
x	5.638 ± 2.75	5.632 ± 2.81	5.699 ± 2.25	0.944
y	10.181 ± 5.58	10.034 ± 5.66	11.503 ± 4.73	0.356
z	5.394 ± 3.78	5.419 ± 3.92	5.171 ± 2.15	0.748
w	16.6 ± 7.72	16.518 ± 8.08	17.34 ± 2.97	0.503
Tibial cut	10.575 ± 4.33	10.66 ± 4.49	9.813 ± 2.3	0.319
Femoral cut	7.175 ± 3.22	7.174 ± 3.3	7.18 ± 2.49	0.994
Post Condyle	8.723 ± 3.77	8.6 ± 3.88	9.826 ± 2.52	0.17
Joint gap	17.822 ± 6.27	17.733 ± 6.53	18.62 ± 3.15	0.449

### Linear regression

**Fig. 5:**
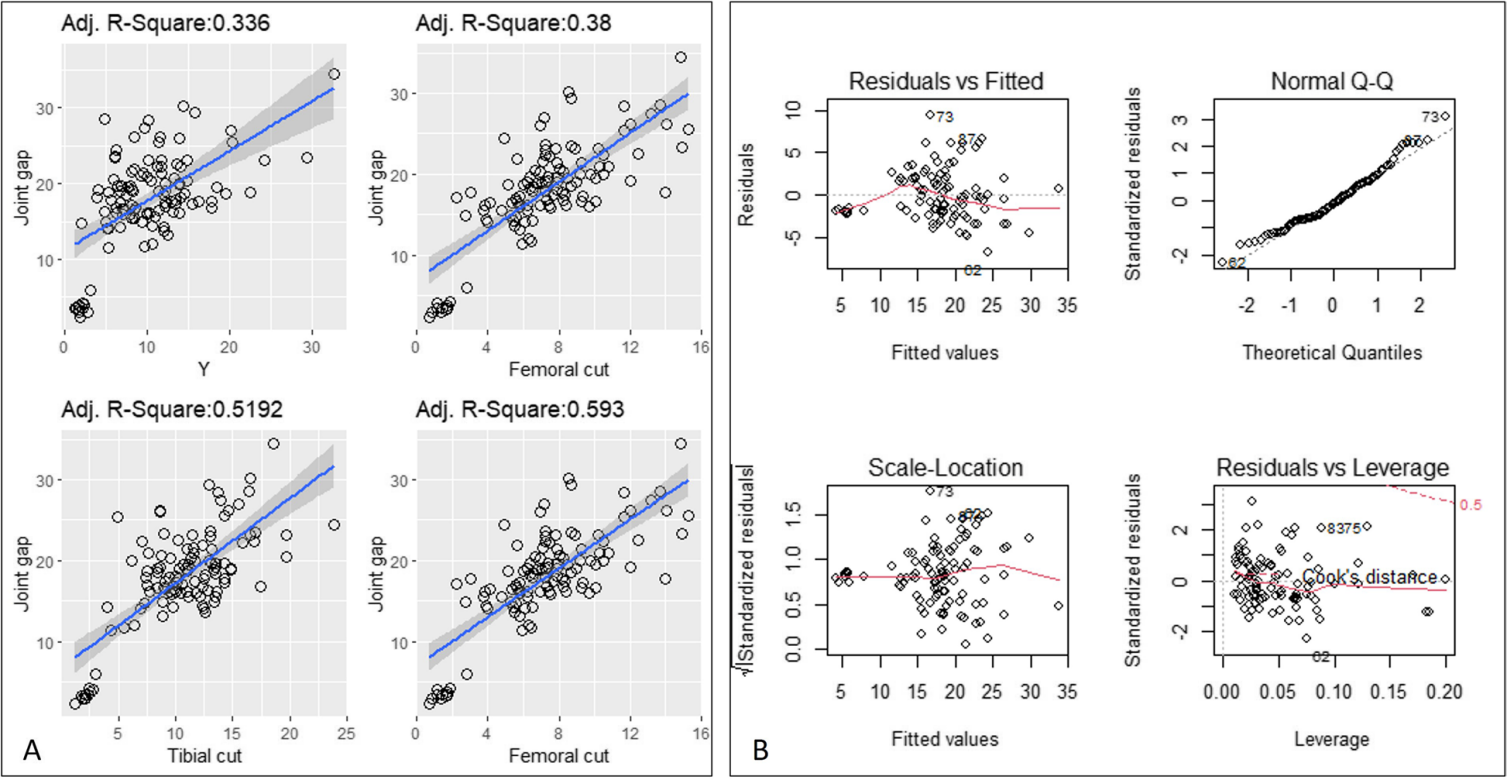
Univariate regression and multivariate regression. **A** The linear relationships between the predictors and Joint Gap. Residual and fitting values (**B**, upper left). Data points between residual and fitting values were evenly distributed on both sides of *y* = 0, showing a random distribution, and the red line presented a smooth curve. Residual Q-Q chart (**B**, upper right), data points were distributed diagonally in a straight line, conforming to normal distribution. Normalized residual square root and fitting value (**B**, bottom left), data points were evenly distributed on both sides of *y* = 0, showing a random distribution. Standardized residuals and leverage values (**B**, bottom right), with no outliers that affected the regression model

Univariate analysis and multivariate analysis results are listed in Table 4. Each predictor had a significant influence on the dependent variable in the univariate analysis (*P* < 0.05). The linear relationships were clearly shown between the predictors and Joint Gap (Fig. 5A).

**Table 4:** Univariate and multivariate analysis

Variables	OR (95%CI)	*P*-value	OR (95%CI)	*P*-value
x	3.609 (2.52–5.17)	< 0.01**		
y	1.931 (1.62–2.98)	< 0.01**	1.162(1.03–1.32)	< 0.01**
z	2.48 (1.89–3.22)	< 0.01**		
w	1.694 (1.51–1.91)	< 0.01**		
Tibial cut	2.855 (2.36–3.45)	< 0.01**	1.736(1.47–2.06)	< 0.01**
Femoral cut	4.499 (3.55–5.69)	< 0.01**	2.593(2.08–3.24)	< 0.01**
Post Condyle	2.809 (2.19–3.6)	< 0.01**	1.217(1–1.48)	< 0.01**

^***^: *P* < 0.001, **: *P* < 0.01, *: *P* < 0.05

Moreover, backwards-stepwise selection was conducted. Considering the collinearity of linearity, y, thickness of tibial cut, femoral cut and post condyle were retained in the multivariate analysis.

Distributions of residual and fitting values were shown in Fig. 5B, and satisfied the linear regression hypothesis.

### The ten-fold cross validation

The result of the tenfold cross-validation is shown in Table 5. The performance of the ten-fold model showed that the R-squared value was large. And the RMSE, MAE values were relatively low, which indicated that the model had good-fitting and was robust.

**Table 5:** Ten-fold cross-validation result of linear regression

Folds	R-Squared	RMSE	MAE
1	0.7909	2.4680	2.2745
2	0.8112	3.413 2	2.1935
**3**^a^	**0.8191**	**3.4761**	**2.1489**
4	0.7757	2.0001	2.3061
5	0.7689	1.3435	2.3787
6	0.8062	3.4278	2.2121
7	0.8102	3.7062	2.1545
8	0.7872	2.7644	2.2485
9	0.7742	2.2820	2.3084
10	0.8006	3.9689	2.1762
All (Mean ± SD)	0.794 ± 0.02	2.8851 ± 0.85	2.2402 ± 0.08

^a^Fold 3 showed the best R-squared value, and was chosen for model establishment

The best performance fold was chosen for the establishment of the best model. R-Squared equaled 0.8191, which was close to 1 in the range of 0 and 1. The RMSE equaled 3.4761, which was acceptable in clinical practice.

Joint Gap calculation equation was as follows:

$$S\left(Joint\;Gap\right)=1.82+0.15*y+0.552*Tibial\;cut+0.953*Femoral\;cut+0.197*Post\;Condyle$$

**Fig. 6:**
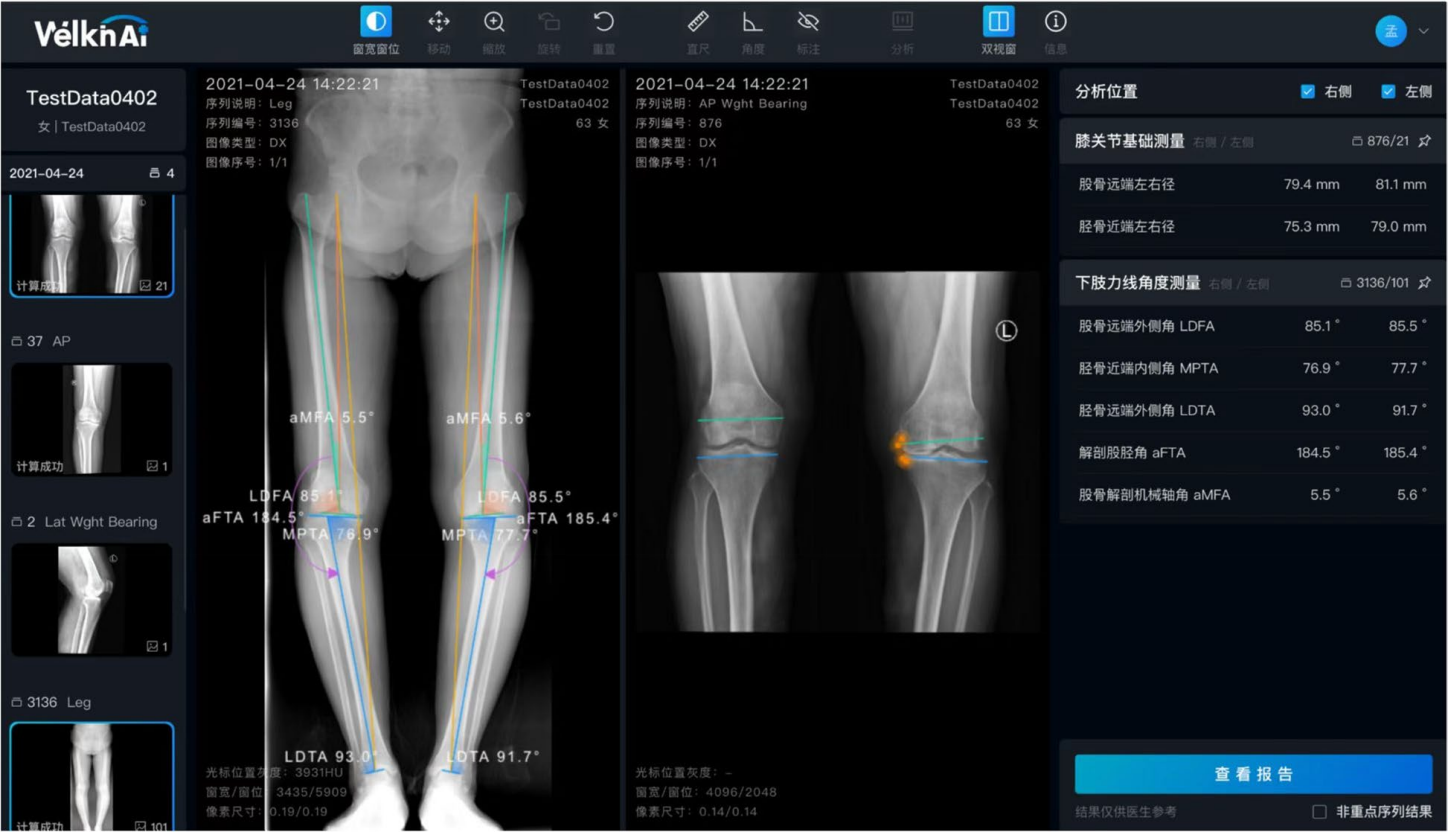
The software based on the predictive equation

According to the aforementioned equation, a 2-D preoperative planning system was developed with adjustable parameters such as planned resection thicknesses and orientations of prosthesis, which may provide a rough prediction of joint gap based on surgeon-preset bone cutting (Fig. 6).

Postoperative parameters were demonstrated on the right panel. Osteophytes were identified automatically, and joint gap was calculated according to the predictive equation and preset bone cuts. Orientation and height of bone cuts can be adjusted.

## Discussion

Preoperative planning is a good start for all surgical procedures. Analog films had been used for years before digital radiographs were introduced. Errors in magnification made planning on analog films inaccurate. Digital preoperative planning has been proven to be more accurate in hip and knee arthroplasties [11, 12]. However, issues, such as implant rotation and extraarticular deformities, remained unaddressed in 2D planning procedure [13–15]. Recently, 3D templating based on CT or MRI scan yielded better accuracy in predicting implant size as compared to traditional 2D films [16]. Meanwhile, Kobayashi et al. demonstrated the unnecessity of applying 3D templating before TKA, as their results failed to support the superiority of the technique in predicting implant size preoperatively [17]. Robotic systems attained better results in alignment in TKA than individualized 3D planning, although the former is time-consuming and doesn’t produce superior PROMs than the latter [18]. Moreover, any additional technique rather than traditional 2D templating may incur additional cost or radiation exposure. We attempted to plan on plain films, with influence on soft tissues taken into account. To our knowledge, it was the first attempt to quantitatively determine soft tissue release on a 2-D planning system.

By using the aforementioned equation, if the amount of preoperative femoral and tibial cut is known, targeted joint gap can be calculated and serve as a reference to surgeons. It should be mentioned that not all dimensions of osteophytes were involved in the equation. Only distance between tips of osteophyte protrusions significantly affected posterior joint gap. Additionally, it is well-known that the amount of bone cut will affect joint gap achieved, and, further, the thickness of tibial insert. This has been taken into account in the building of the model.

Machine learning has been widely used in medical fields and for preoperative planning of TKA, including implant size, position, economic analysis, outcome evaluation and patient/implant follow-ups [19–24]. Cross-validation is a method used for model and data-set validation to estimate the out-of-sample error. It has become quite popular because of its simplicity and utility [25, 26]. Ten-fold cross-validation performs the fitting procedure a total of ten times, with each fit being performed on a training set consisting of 90% of the total training set selected at random, with the remaining 10% used as a holdout set for validation. In our study, we conducted a ten-fold cross-validation by using the R software to avoid sampling bias and it is an effective attempt to use the machine learning concept to deal with large samples.

Our study is subject to limitations. First, all data were reviewed retrospectively, while the sample size was relatively small. Loss of intraoperative information might bring sample bias to the study. We conducted a cross-validation of samples to minimize the influence of sample size. Secondly, Some factors, such as surgeons' understanding about soft tissue balancing that might influence their maneuver or mis-handlings during operation, are not taken into consideration. Moreover, surgeons' preference for balancing in TKA does influence the establishment of the model. We attempted to adjust parameters in the system to accommodate different surgical preferences such as ligament balancing or measurement resection in the published edition of our software. A prospective study with better design may be closer to real-world practice, and will improve the accuracy of planning. Implant variables were not considered in the model, either, because minimal variation in implant dimensions might not influence the results significantly. Last but not least, the equation we worked out may be useful in guiding preoperative planning but uncertainties in surgical procedures require the ability to respond flexibly during operation. A surgeon should take all possible factors into account preoperatively in order to perform a perfect surgery.

## Conclusion

In this study, we have worked out an equation to predict the influence of preoperative radiographic elements on the soft tissue balancing before TKA, and a preoperative planning system has been developed. The computer-assisted method was proven useful in generating a reliable equation. Further studies are warranted to modify and improve the model to achieve better results.

## Data Availability

Study data and material were held by researchers in the Chinese PLA General Hospital. The datasets used and analyzed during the current study are available from the corresponding author upon reasonable request.
